# Uncoordinated expression of DNA methylation-related enzymes in human cancer

**DOI:** 10.1186/s13072-017-0170-0

**Published:** 2017-12-12

**Authors:** Jiao Liu, Xiuliang Cui, Jinhua Jiang, Dan Cao, Yufei He, Hongyang Wang

**Affiliations:** 1 State Key Laboratory of Oncogenes and Related Genes, Shanghai Cancer Institute, Renji Hospital, Shanghai Jiao Tong University School of Medicine, Shanghai, People’s Republic of China; 2 International Cooperation Laboratory on Signal Transduction, Eastern Hepatobiliary Surgery Institute/Hospital, 225 Changhai Road, Shanghai, 200438 People’s Republic of China; 3 National Center for Liver Cancer, Shanghai, 201805 People’s Republic of China

**Keywords:** DNA methylation-related enzymes, Correlation, 5mC, 5mC oxidation derivatives

## Abstract

**Background:**

In addition to the important roles played by 5-methylcytosine (5mC), emerging evidence suggests that 5mC derivatives, such as 5-hydroxymethylcytosine (5hmC), 5-formylcytosine (5fC) and 5-carboxylcytosine (5caC), also exhibit regulatory functions in physiological and pathological processes. Four cytosine modifications (5mC, 5hmC, 5fC and 5caC) are produced and erased by a cyclic enzymatic cascade mediated by DNA methyltransferases (DNMTs), ten-eleven translocation (TET) family enzymes and thymine DNA glycosylase (TDG). Stable maintenance of the DNA methylation profile is important for normal cell homeostasis, but its underlying mechanisms are largely unknown.

**Methods:**

The expression levels of 7 DNA methylation-related enzymes from normal mouse tissues were assessed using quantitative real-time RT-PCR (qRT-PCR). The gene expression data and related information of human normal tissues and tumor tissues were obtained from the Genotype-Tissue Expression (GTEx) and the Cancer Genome Atlas (TCGA), respectively.

**Results:**

We observed significant positive correlations among the expression levels of DNA methylation-related enzymes in various mice and human normal tissues. By contrast, we found significantly decreased correlations in various tumor tissues compared with their corresponding normal tissues. Furthermore, we also found that alterations in these correlations are associated with several clinicopathological characteristics of cancer patients.

**Conclusions:**

These observations suggest that uncoordinated expression of DNA methylation-related enzymes is another epigenetic hallmark of cancer. Our work provides important insights into an additional regulatory layer of the DNA methylation maintenance machinery.

**Electronic supplementary material:**

The online version of this article (10.1186/s13072-017-0170-0) contains supplementary material, which is available to authorized users.

## Background

DNA methylation plays essential roles in regulating gene expression in both normal development and diseases [1, 2]. After the establishment of methylation marks by DNA methyltransferases 3A and 3B (DNMT3A and DNMT3B) and maintenance by DNMT1 [3–8], 5-methylcytosine (5mC) can be successively oxidized to 5-hydroxymethylcytosine (5hmC), 5-formylcytosine (5fC) and 5-carboxylcytosine (5caC) by the ten-eleven translocation (TET) family enzymes; thereafter, 5fC and 5caC can be excised and repaired by thymine DNA glycosylase (TDG) in conjunction with the base excision repair pathway to regenerate unmodified cytosine [8–13]. In addition to this pathway for active DNA demethylation, there is a passive DNA demethylation pathway characterized with loss of DNA modifications by replication and cell division [8].

Recently, growing evidence suggests that 5mC and its derivatives can recruit unique proteins with specific functions that may be coupled with gene expression [14–17]. Thus, it is important to properly maintain the levels of 5mC and its derivatives for normal cell homeostasis. However, the mechanisms underlying the maintenance of DNA methylation profiles are largely unknown.

Aberrant DNA methylation is considered to be as an epigenetic hallmark of various types of diseases, including cancer [18]. It has been shown that cancer cells exhibit abnormal DNA methylation profiles characterized by global hypomethylation and focal hypermethylation [19]. Recently, reduced 5hmC has also been reported in human cancers, including kidney tumors, acute myeloid leukemia and liver cancer [20–22].

The alteration and maintenance of the DNA methylation status are directly regulated by DNA methyltransferases and DNA demethylases (a group of enzymes including Tet family enzymes and TDG). However, whether these functionally related enzymes are expressed coordinately and whether the expression patterns of these enzymes are consistent in normal and cancer tissues have not yet been studied.

Here, we experimentally and bioinformatically show that significant positive correlations among the expression levels of DNA methylation-related enzymes are present within normal mouse and human tissues. By contrast, in cancer tissues, these correlations are not so positive.

## Results

### The correlations among DNA methylation-related enzymes are extensive and significantly positive in normal mouse tissues

**Fig. 1:**
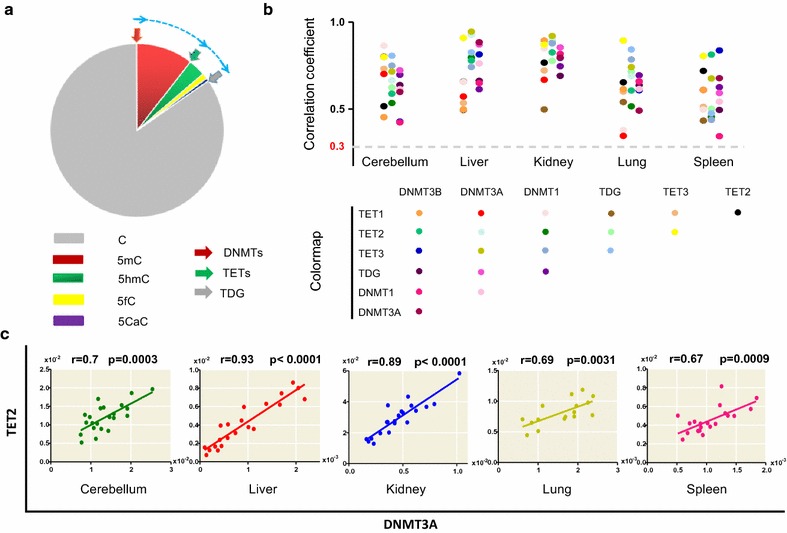
Significant positive correlations among DNA methylation-related enzymes in 5 normal mouse tissues. **a** Schematic overview of the cyclic distribution of different forms of cytosine. After establishment by DNA methyltransferases (DNMTs), 5mC undergoes stepwise oxidation to 5hmC, 5fC and 5caC via the TET family enzymes (TETs), and 5fC and 5caC can be then replaced with unmodified cytosine by TDG in conjunction with the base excision repair pathway (not shown). **b** The value of the correlations between each DNA methylation-related enzyme pair in 5 normal mouse tissues is shown. The balls in different colors represent the Pearson correlation coefficients among the expression levels of 7 DNA methylation-related enzymes (one ball refers to one enzyme pair). The DNA methylation-related enzyme pairs are indicated below. In this study, when the correlation coefficient r was higher than 0.3 (dotted line), the correlation was significantly positive (*p* < 0.05). The detailed Pearson correlation coefficient values and their corresponding *p* values are shown in Additional file 2: Table S1. **c** The correlations between DNMT3A and TET2 in 5 normal mouse tissues are shown. For each tissue type, the normalized mRNA levels of TET2 (*y*-axis) versus the normalized mRNA levels of DNMT3A (*x*-axis) are shown (one ball refers to one sample). The Pearson correlation coefficient and *p* value are also shown

We first explored the correlations among the expression levels of 7 DNA methylation-related enzymes in 5 normal tissue types (liver, cerebellum, kidney, spleen and lung) from 21 wild-type mice. The enzymes we analyzed (Fig. 1a) include direct DNA methylation enzymes (writers, namely, DNMT1, DNMT3A and DNMT3B) and direct DNA demethylation enzymes (erasers, namely, TET1, TET2, TET3 and TDG). The expression levels of these 7 DNA methylation-related enzymes were assessed using quantitative real-time RT-PCR (qRT-PCR). Strikingly, we observed significant positive correlations between almost all DNA methylation-related enzyme pairs in all five tissues (Fig. 1b). Recently, the cooperation and competition between DNMT3A and TET2 and their ability to maintain methylation in hematopoietic stem cells have been reported [23]. Interestingly, our data also showed significant positive correlations between these two DNA methylation-related enzymes in all 5 mouse tissues (Fig. 1c). It should be noted that positive correlations are present among all DNA methylation-related enzymes (Fig. 1, Additional file 1: Figure S1 and Additional file 2: Table S1) rather than just between the aforementioned “writers” and “erasers,” implying that cooperation among DNA methylation-related enzymes may be more prevalent than one might have expected.

### The correlations among DNA methylation-related enzymes are significantly positive in various human tissues

**Fig. 2:**
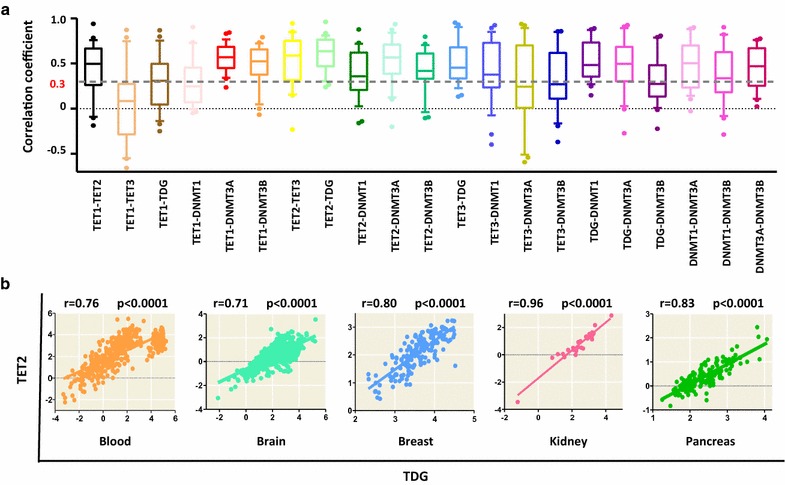
Significant positive correlations among DNA methylation-related enzymes in 26 normal human tissues. **a** Box plots (box and whiskers, 10–90%) illustrating the distribution of the correlation values among the expression levels of 21 DNA methylation-related enzyme pairs in 26 normal human tissues (one ball refers to on tissue). The detailed Pearson correlation coefficients values and their corresponding *p* values are shown in Additional file 4: Table S2. **b** The correlations between TET2 and TDG expression levels in 5 normal human tissues are shown. For each tissue type, the normalized mRNA levels of TET2 (*y*-axis) versus the normalized mRNA levels of TDG (*x*-axis) are shown (one ball refers to one sample). The Pearson correlation coefficient and *p* value are also shown

To investigate whether the aforementioned positive correlations were also present in normal human tissues, we downloaded expression data of the 7 genes in 31 human tissues from the Genotype-Tissue Expression (GTEx) project. Three tissues, including the bladder, cervix uteri and fallopian tube, were excluded due to their limited number of samples (there were only 9, 11 and 7 samples, respectively); additionally, the testis and ovary were excluded because gametes possess unique DNA methylation profiles [8]. The final 26 tissues used in this study are listed in Additional file 4: Table S2. The number of samples analyzed in each tissue ranged from 27 (kidney) to 1146 (brain). A total of 7554 tissue samples were analyzed (Additional file 4: Table S2). We found significant positive correlations among DNA methylation-related enzymes in nearly all 26 tissues (Fig. 2a and Additional file 4: Table S2). In particular, the correlations between TET2 and TDG; TET1 and DNMT3A; and TDG and DNMT1 were stronger than the others (Fig. 2a, b, Additional file 3: Figure S2 and Additional file 4: Table S2). In addition, the correlations in the blood, brain, breast, kidney and pancreas were stronger than those in other tissues (Fig. 2b and Additional file 4: Table S2).

We also analyzed the expression data of the 7 genes in testis and ovary tissues which possess unique DNA methylation profiles [8]. Interestingly, some correlations (for example, TET1-DNMT1 and TET2-DNMT1 in both tissues) were significantly negative (Additional file 5: Table S3), although most of the correlations were still significantly positive.

To exclude the possibility that the observed positive correlations between the DNA methylation-related enzymes were just the overall transcriptional activity that was measured in GTEx, we also analyzed the expression level of other 3 unrelated genes: β-actin (ACTB), ADH1A and CYP4B1. We first normalized the expression data of the 7 DNA methylation-related genes, ADH1A and CYP4B1 from GTEx to β-actin mRNA expression. We found that the overall results of the correlation between the DNA methylation-related genes were improved after normalization, while the correlations between ADH1A or CYP4B1 and DNA-related enzymes were largely not significant in most of tissues (Additional file 6: Table S4). Taken together with the mouse results obtained from the real-time PCR data (Fig. 1, Additional file 1: Figure S1 and Additional file 2: Table S1), we conclude that correlations among DNA methylation-related enzymes are significantly positive in various normal mouse and human tissues.

### Lower correlations among the expression levels of DNA methylation-related enzymes in human cancer tissues

**Fig. 3:**
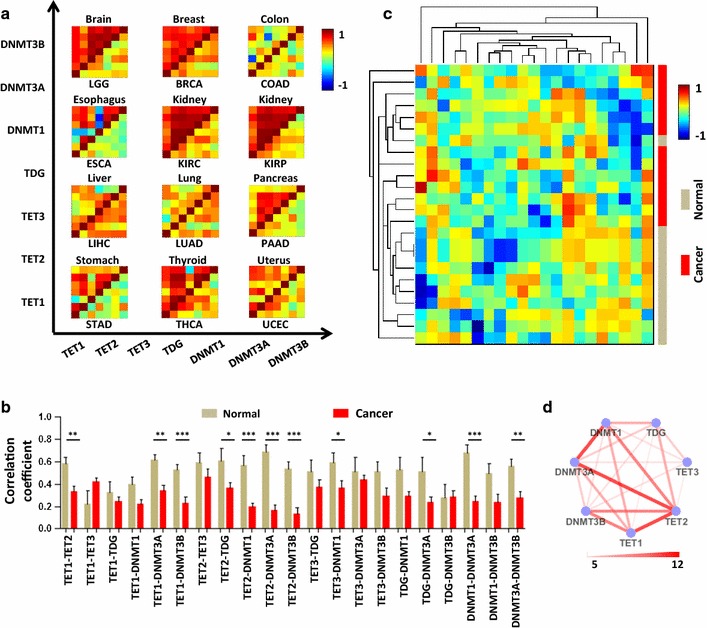
Lower correlations among the expression levels of 7 DNA methylation-related enzymes in human cancer tissues. **a** Heat map of the correlations of gene expression levels among 7 DNA methylation-related enzymes in 12 normal tissues (upper triangle) and the corresponding tumor tissues (lower triangle). LGG: brain lower-grade glioma; BRCA: breast invasive carcinoma; COAD: colon adenocarcinoma; ESCA: esophageal carcinoma; KIRC: kidney renal clear cell carcinoma; KIRP: kidney renal papillary cell carcinoma; LIHC: liver hepatocellular carcinoma; LUAD: Lung adenocarcinoma; PAAD: pancreatic adenocarcinoma; STAD: stomach adenocarcinoma; THCA: thyroid carcinoma; and UCEC: uterine corpus endometrial carcinoma. **b** Bar plot of the correlations among the expression levels of 21 gene pairs in normal and tumor tissues. *p < 0.05, **p < 0.01 and ***p < 0.001 by paired *t* test. **c** Cluster analysis based on the correlation levels of 21 gene pairs (*x*-axis) in 12 normal and tumor tissues (*y*-axis). Each square represents one out of 21 correlations in one normal or tumor tissue. **d** The connectivity map of 7 genes; the edge thinness and color represent the number of tissue types in which the correlation of the gene pair is higher in the normal tissue than in the tumor tissue

Given the positive correlations among DNA methylation-related enzymes in normal tissues and the abnormal 5mC and 5hmC content in cancer [19–22], we next asked whether the correlations among the expression levels of DNA methylation-related enzymes are altered in cancer tissues. Expression data of the 7 genes in normal and tumor tissues were obtained from the Genotype-Tissue Expression (GTEx) and the Cancer Genome Atlas (TCGA) databases. Data on twelve solid tissues from both databases were evaluated in our study. The correlations among the 7 genes in normal and tumor tissues were calculated. As shown in Fig. 3a, b, the 7 genes were less correlated in tumor tissues than in normal tissues, which was observed in 10 tissue pairs out of 21 pairs (Fig. 3a), and the difference was significant in 11 DNA methylation-related enzyme pairs (Fig. 3b). Unsupervised clustering was performed, and we found that the correlations of the gene expression levels among the 7 DNA methylation-related enzymes had distinct patterns between normal tissues and tumor tissues (Fig. 3c). The network showed that the top 3 most different correlations between normal and tumor tissues among all 21 DNA methylation-related enzyme pairs were DNMT1-DNMT3A, TET2-DNMT3A and TET1-TET2 (Fig. 3d).

Additionally, given the most notable DNA methylation effects in leukemia [21], we also compared specially these correlations between in blood and leukemia. We found that the correlations among DNA methylation-related enzymes in leukemia were significantly lower or even negative (Additional file 7: Table S5).

**Fig. 4:**
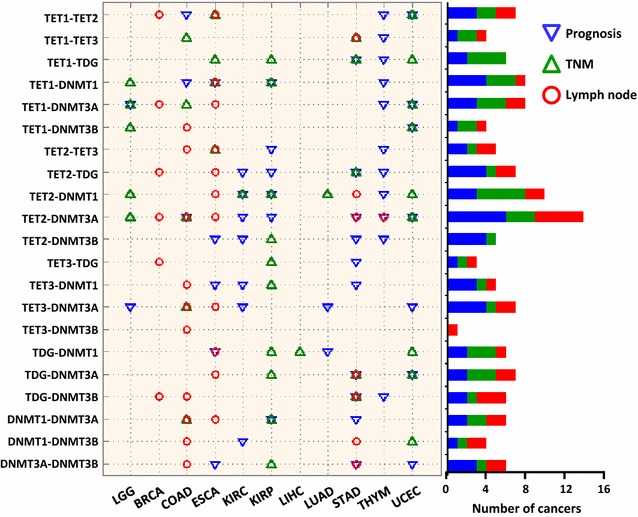
Association between the correlations of 21 DNA methylation-related enzyme pairs and clinical characteristics. A blue del indicates that the difference in the correlations of the DNA methylation-related enzyme pairs in early recurrence patients and non-early recurrence patients is greater than 0.15. Similarly, a green triangle and a red circle represent the TNM stage and the lymph node examined, respectively. The bar plot (right) shows the total number of cancer types with significant difference in the correlations across the 3 clinical characteristics

We further investigated whether the changed correlation level was associated with the clinical characteristics of cancer patients. We focused on 3 important clinical characteristics: recurrence; tumor, node and metastasis (TNM) staging; and the lymph node examined. Patients were classified into 2 groups according to the 3 clinical characteristics, respectively, and the difference of the correlation between the DNA methylation-related enzymes in the 2 groups was calculated. (Detailed information is in “Methods” section.) As shown in Fig. 4, the differences in the correlations between several DNA methylation-related enzyme pairs, especially TET2-DNMT3A and TET2-DNMT1, were related to these 3 clinical characteristics across various cancer types.

## Discussion

Here, we experimentally and bioinformatically proved the presence of extensive and significant positive correlations among DNA methylation-related enzymes in normal mouse and human tissues. This is consistent with the need for these enzymes to not only functionally cooperate but also functionally compete, and these relationships are likely required to properly maintain relatively stable levels of 5mC and its derivatives. More importantly, we also observed significantly decreased correlations among DNA methylation-related enzymes in cancer tissues.

The exact function of oxidized 5mC derivatives remains elusive; however, it is increasingly clear that the oxidized forms of 5mC represent important dynamic epigenetic states in the modulation of transcriptional programs and serve as signals for several specific “binders,” “readers” or “erasers.” It has been demonstrated that 5mC and its derivatives recruit distinct transcriptional regulators, polymerases and a large number of DNA repair proteins [14–17]. Even a single copy of 5fC can markedly increase DNA flexibility [24]. Conceivably, maintenance of the appropriate levels of 5mC and its derivatives is essential for cell homeostasis. Indeed, the DNA methylation profile is stably maintained in somatic cells [8, 25, 26]; however, its underlying mechanisms are still elusive.

Since the total amount of cytosine (modified and unmodified cytosine) is constant, and its cyclic conversion to different modification states is achieved by an enzymatic cascade of DNA methylation-related enzymes (Fig. 1a), the cyclic distribution of different forms of cytosine is cooperatively and competitively determined by the enzymatic activities of DNA methylation-related enzymes. An influential model of maintenance methylation is that DNA methylation at CpG sites is maintained by the specific activity of DNMT1 enzymes [8]. However, a revised model proposed that the DNA methylation at each site is maintained by DNMT enzyme, TET enzymes and the DNA replication rate [27].

It has been shown that many factors can influence the catalytic activities of DNA methylation-related enzymes, such as chemical modifications, cellular metabolites and cofactors [8, 28–31]. Regardless, the expression of DNA methylation-related enzymes represents a fundamental layer of their methylation maintenance regulatory mechanisms. Thus, we investigated whether there are correlations at the transcriptional level among DNA methylation-related enzymes.

Consistently, we found the presence of extensive and significant positive correlations among DNA methylation-related enzymes in various normal mouse and human tissues. Of note, we found that there were positive correlations not only between the expression levels of the “writers” and “erasers” (DNMTs and TETs or TDG and DNMTs) but also among DNMT enzymes and among TET enzymes. Our data may provide insights into an alternative manner of cooperation and competition at the transcriptional level among DNA methylation-related enzymes in methylation maintenance. Our findings may also provide additional evidence for a recently proposed modified DNA maintenance methylation model [27].

Aberrant DNA methylation characterized by global hypomethylation and focal hypermethylation has long been considered as an epigenetic hallmark of cancer cells [18]. Although it has been demonstrated that loss of or mutations in DNMTs and/or TETs are associated with aberrant DNA methylation [23, 32, 33], the detailed mechanisms are still not well understood.

In this work, we observed uncoordinated expression levels of DNA methylation-related enzymes in various types of cancer tissues. These observations suggest that aberrant DNA methylation profiles in cancers may be partly due to altered cooperativity among DNA methylation-related enzymes in addition to the deregulation of a specific enzyme. Additionally, we also observed that the decreased correlations were associated with several clinicopathological characteristics and diagnostic markers, as evidenced in various types of cancers (Fig. 4). Taken together, our results indicate that the uncoordinated expression of DNA methylation-related enzymes is another epigenetic hallmark of cancer. Additional studies will be required to better understand the underlying mechanisms of the extensive positive correlations among DNA methylation-related enzymes and to understand how the altered correlations are associated with the clinicopathological characteristics and prognosis of cancer.

## Conclusions

Our data not only provide important insights into an additional regulatory layer of the DNA modification maintenance machinery but also demonstrate that the positive correlations among DNA methylation-related enzymes are disrupted in cancer cells, which may contribute to aberrant DNA methylation. Moreover, we found associations between the aforementioned altered correlations and the clinicopathological characteristics of patients. These findings suggest that uncoordinated expression of DNA methylation-related enzymes is another epigenetic hallmark of cancer.

## Methods

### Mouse tissue specimens

Cerebellum, kidney, spleen, lung and liver tissues were collected from 21 wild-type Balb/c mice (8–10 weeks old). The mice were obtained from the Chinese Science Academy in Shanghai, China. The study was approved by the Eastern Hepatobiliary Surgery Hospital Ethics Committee.

### Gene expression analysis

Total RNA was isolated from tissues with TRIzol reagent (Invitrogen). After DNase I treatment, complementary DNA was synthesized according to the manufacturer’s instructions (Takara). qRT-PCR was carried out using SYBR Green Mix according to the manufacturer’s instructions (Roche). Expression data were normalized to β-actin mRNA expression. The obtained data are presented in arbitrary units and were calculated using the formula: 2^(− ΔCt^ ^β-actin − gene of interest)^. The sequences of the primers are listed in Additional file 8: Table S6.

### Datasets

The expression data of the 7 genes across tissue types were obtained from the Genotype-Tissue Expression (GTEx) Project [34] (normal human tissues) and TCGA database [35] (tumor samples). The normalized gene-level RNA-Seq data were downloaded from UCSC Xena (http://xena.ucsc.edu/). The clinical information of tumor patients was accessed from the TCGA public access web portal (https://cancergenome.nih.gov/).

### Clinical characteristics relationship analysis

To investigate the potential roles of the correlations among the 7 genes in cancer development, we studied the relationship between the gene correlations and clinical characteristics of cancer patients, and 3 important and common clinical characteristics were analyzed in 11 cancers. For recurrence analyses, we divided the patients into two groups for each cancer type. Patients who were dead or recurrence within 3 years were placed into the early group, and the remaining patients were placed into the non-early recurrence group. For the TNM staging analysis, patients with TNM stages I and II were classified into group 1, and patients with TNM stages III and IV were classified into group 2. For lymph node analyses, lymph node-positive patients were classified into group 1, and lymph node-negative patients were classified into group 2. The correlations among the 7 genes between the two groups were determined.

### Statistical analysis

The correlations among the expression levels of DNA methylation-related enzymes were assessed by Pearson’s correlation analysis. A value of *p* < 0.05 after multiple-testing correction using Benjamini–Hochberg procedure was considered statistically significant. All analyses were performed using the professional statistical software GraphPad Prism version 5.01.

## Additional files

**Additional file 1: Figure S1.** Scatter plots of 21 DNA methylation-related enzyme pairs in 5 normal mouse tissues are shown. For each enzyme pair, the normalized mRNA levels of two genes in each tissue are plotted and are distinguished by different colors.

**Additional file 2: Table S1.** Correlations among DNA methylation-related enzymes in 5 mouse tissues. The expression of 7 DNA methylation-related enzymes in 5 mouse tissues was quantified by real-time qRT-PCR. The correlations between these enzymes are analyzed and shown. A multiple-testing correction was done by using Benjamini–Hochberg procedure. The corrected *p* value is shown only when the *p* value was changed from < 0.05 to > 0.05 after the correction.

**Additional file 3: Figure S2.** The correlations between TET2 and TDG in 26 normal human tissues are shown. For each tissue type, the normalized mRNA levels of TET2 (*y*-axis) versus the normalized mRNA levels of TDG (*x*-axis) are shown. The Pearson correlation coefficient and *p* value are also shown. Ad: adipose tissue; Ag: adrenal gland; BV: blood vessel; BM: bone marrow; SG: salivary gland; and SI: small intestine.

**Additional file 4: Table S2.** Correlations among DNA methylation-related enzymes in 26 human tissues. The RNA-Seq gene expression data of 7 DNA methylation-related enzymes were obtained from the GTEx dataset. The correlations among the expression levels of the 7 enzymes in 26 human tissues are analyzed and shown. A multiple-testing correction was done by using Benjamini–Hochberg procedure. The corrected *p* value is shown only when the *p* value was changed from < 0.05 to > 0.05 after the correction.

**Additional file 5: Table S3.** Correlations among DNA methylation-related enzymes in testis and ovary tissues. The RNA-Seq gene expression data of 7 DNA methylation-related enzymes were obtained from the GTEx dataset. The correlations among the expression levels of the 7 enzymes are analyzed and shown.

**Additional file 6: Table S4.** Correlations among DNA methylation-related enzymes, ADH1A and CYP4B1 after normalization. The RNA-Seq gene expression data were obtained from the GTEx dataset and normalized to β-actin mRNA expression. The correlations among the expression levels of the 7 DNA methylation-related enzymes as well as between ADH1A or CYP4B1 and DNA methylation-related enzymes in 26 human tissues are analyzed and shown. A multiple-testing correction was done by using Benjamini–Hochberg procedure. The corrected *p* value is shown only when the *p* value was changed from < 0.05 to > 0.05 after the correction.

**Additional file 7: Table S5.** Correlations among DNA methylation-related enzymes in blood and leukemia. The RNA-Seq gene expression data of 7 DNA methylation-related enzymes were obtained from the GTEx and TCGA dataset. The correlations among the expression levels of the 7 enzymes are analyzed and shown.

**Additional file 8: Table S6.** Primer pairs for real-time PCR.

## Abbreviations

5mC: 5-methylcytosine
5hmC: 5-hydroxymethylcytosine
5fC: 5-formylcytosine
5caC: 5-carboxylcytosine
DNMTs: DNA methyltransferases
TET: ten-eleven translocation
TDG: thymine DNA glycosylase
GTEx: the Genotype-Tissue Expression
TCGA: the Cancer Genome Atlas
qRT-PCR: quantitative real-time RT-PCR
TNM: tumor, node and metastasis
