# Supplementary figures and images for: Uncoordinated expression of DNA methylation-related enzymes in human cancer
Source: Epigenetics Chromatin. 2017 Dec 12;10:61. doi: 10.1186/s13072-017-0170-0 (PMC5727647; doi:10.1186/s13072-017-0170-0)

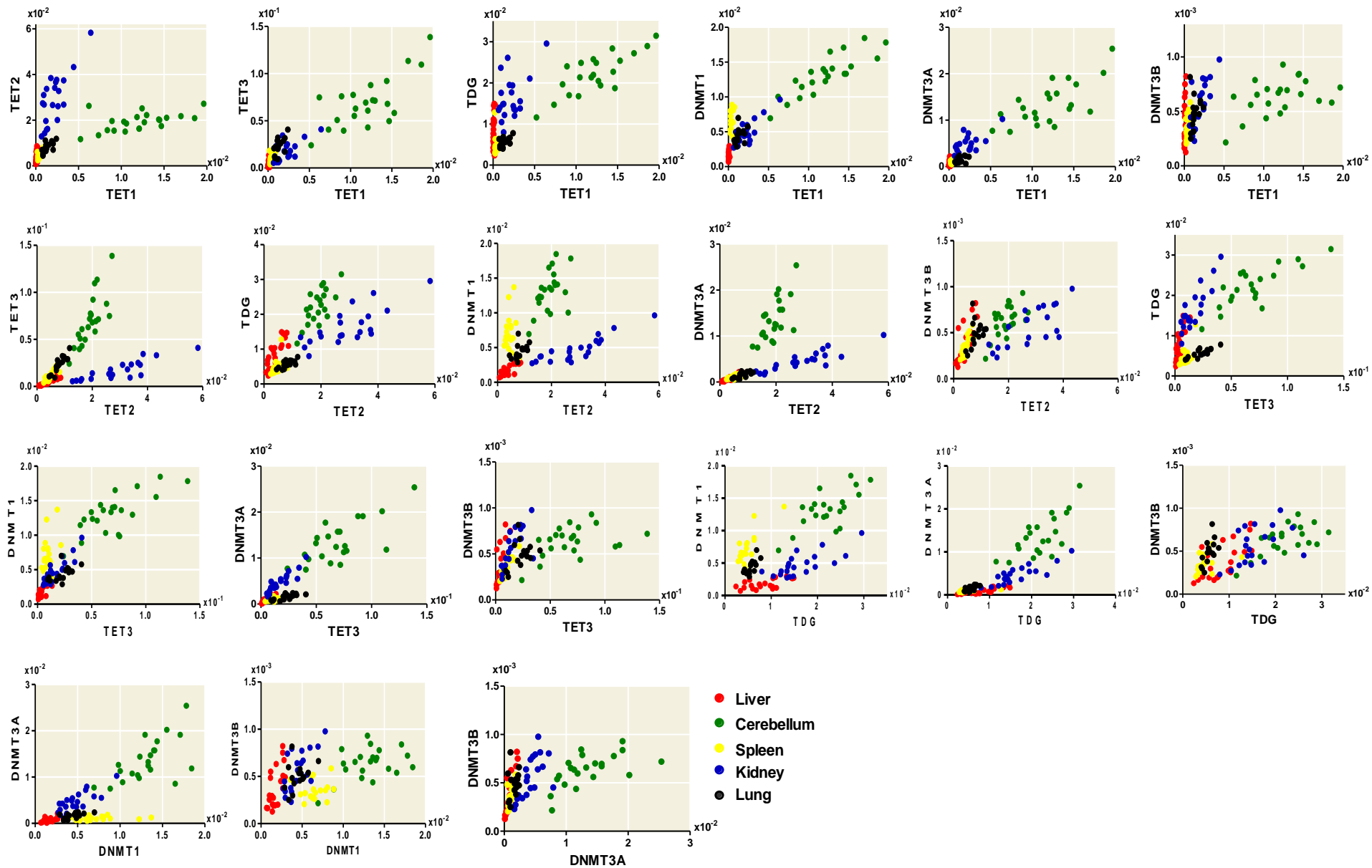

Supplement: Supplementary file 1 — Additional file 1: Figure S1. Scatter plots of 21 DNA methylation-related enzyme pairs in 5 normal mouse tissues are shown. For each enzyme pair, the normalized mRNA levels of two genes in each tissue are plotted and are distinguished by different colors. [file 13072_2017_170_MOESM1_ESM.pdf]

TET2

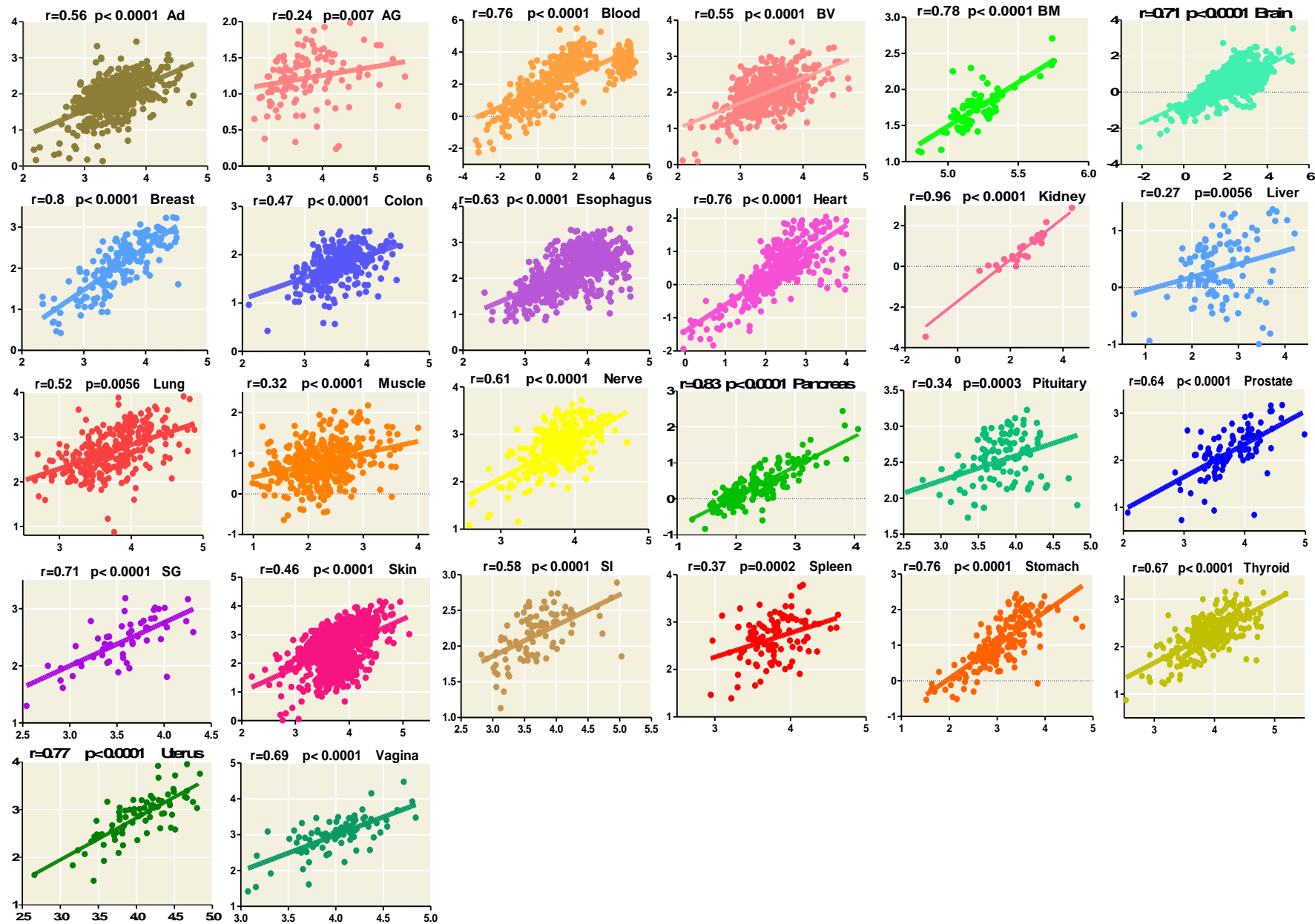

TDG

Supplement: Supplementary file 3 — Additional file 3: Figure S2. The correlations between TET2 and TDG in 26 normal human tissues are shown. For each tissue type, the normalized mRNA levels of TET2 (y-axis) versus the normalized mRNA levels of TDG (x-axis) are shown. The Pearson correlation coefficient and p value are also shown. Ad: adipose tissue; Ag: adrenal gland; BV: blood vessel; BM: bone marrow; SG: salivary gland; and SI: small intestine. [file 13072_2017_170_MOESM3_ESM.pdf]
