# Supplementary material for: Uncoordinated expression of DNA methylation-related enzymes in human cancer
Source: Epigenetics Chromatin. 2017 Dec 12;10:61. doi: 10.1186/s13072-017-0170-0 (PMC5727647; doi:10.1186/s13072-017-0170-0)
Supplement: Supplementary file 8 — Additional file 8: Table S6. Primer pairs for real-time PCR. [file 13072_2017_170_MOESM8_ESM.docx]

**List of primer pairs for real-time PCR in mice tissues**

| **Gene name** | **Primer seqence** |
| --- | --- |
| mTET1 | 5^，^-CATTCTCACAAGGACATTCACAACA-3**^,^**  5^，^-AGTAAAACGTAGTCGCCTCTTCCTG-3**^,^** |
| mTET2 | 5^，^-GACTGCTGTTTGGGTCTGAAGG-3**^,^**  5^，^-TGACCACCACTGTACTGCCATT-3**^,^** |
| mTET3 | 5^，^-AGGCAGCTAAGCACCTCAG-3**^,^**  5^，^--GGCCCCGTAAGATGACACAG-3**^,^** |
| mTDG | 5^，^-AAGTACGGCATCGGATTCAC-3**^,^**  5^，^-TTCTGCAGTTTCTGCACCAG-3**^,^** |
| mDNMT1 | 5^，^-GTGCCCTGCGTGGAGTCTGT-3**^,^**  5^，^-GTGGTTGTGCCGGTTCCCAGTG-3**^,^** |
| mDNMT3A | 5^，^-CGACTGCGAGGTGGCTTGGGCTG-3**^,^**  5^，^-CTCCACCTTCTGAGACTCTCCAGAG-3**^,^** |
| mDNMT3B | 5^，^-TTCAGTGACCAGTCCTCAGACACGAA-3**^,^**  5^，^-TCAGAAGGCTGGAGACCTCCCTCTT-3**^,^** |
| mß-actin | 5^，^-GAAATCGTGCGTGACATCAAAG-3**^,^**  5^，^-TGTAGTTTCATGGATGCCACAG-3**^,^** |
|  |  |
